# Electroencephalographic Parameters Differentiating Melancholic Depression, Non-melancholic Depression, and Healthy Controls. A Systematic Review

**DOI:** 10.3389/fpsyt.2021.648713

**Published:** 2021-08-19

**Authors:** Caroline Fussing Bruun, Caroline Juhl Arnbjerg, Lars Vedel Kessing

**Affiliations:** ^1^Copenhagen Affective Disorder Research Center (CADIC), Psychiatric Center Copenhagen, Copenhagen, Denmark; ^2^Department of Public Health, Center for Global Health, Aarhus University, Aarhus, Denmark; ^3^Faculty of Health and Medical Sciences, University of Copenhagen, Copenhagen, Denmark

**Keywords:** melancholic depression, subtypes of depression, major depressive disorder, electroencephalography, biomarkers

## Abstract

**Introduction:** The objective of this systematic review was to investigate whether electroencephalographic parameters can serve as a tool to distinguish between melancholic depression, non-melancholic depression, and healthy controls in adults.

**Methods:** A systematic review comprising an extensive literature search conducted in PubMed, Embase, Google Scholar, and PsycINFO in August 2020 with monthly updates until November 1st, 2020. In addition, we performed a citation search and scanned reference lists. Clinical trials that performed an EEG-based examination on an adult patient group diagnosed with melancholic unipolar depression and compared with a control group of non-melancholic unipolar depression and/or healthy controls were eligible. Risk of bias was assessed by the Strengthening of Reporting of Observational Studies in Epidemiology (STROBE) checklist.

**Results:** A total of 24 studies, all case-control design, met the inclusion criteria and could be divided into three subgroups: Resting state studies (*n* = 5), sleep EEG studies (*n* = 10), and event-related potentials (ERP) studies (*n* = 9). Within each subgroup, studies were characterized by marked variability on almost all levels, preventing pooling of data, and many studies were subject to weighty methodological problems. However, the main part of the studies identified one or several EEG parameters that differentiated the groups.

**Conclusions:** Multiple EEG modalities showed an ability to distinguish melancholic patients from non-melancholic patients and/or healthy controls. The considerable heterogeneity across studies and the frequent methodological difficulties at the individual study level were the main limitations to this work. Also, the underlying premise of shifting diagnostic paradigms may have resulted in an inhomogeneous patient population.

**Systematic Review Registration:** Registered in the PROSPERO registry on August 8th, 2020, registration number CRD42020197472.

## Introduction

Melancholic depression, a subtype of unipolar depression characterized by neurovegetative symptoms, anhedonia, and weakened emotional reactivity, has been a central syndrome in especially European psychiatric tradition and remains today, although ongoing discussions about its validity as a separate diagnostic entity, decidedly clinically relevant. Depression with melancholic features is preserved as a specifier in DSM-5 (1), as well as in ICD-11 (2). Whereas, translation across diagnostic paradigms is never without complications, it has largely replaced the former designation “endogenous depression.” While its pathophysiological underpinnings have been explored for decades, no clinically applicable biomarkers are available to support today's purely descriptive diagnoses.

One early established pathway was the attempt to identify abnormal neurophysiological patterns underlying the melancholic symptomatology; structural or functional brain alterations due to mood disorder were hypothesized to alter the neuronal oscillations detectable by electroencephalography (EEG). For more than four decades, there has been researched extensively in the field of EEG and mood disorder, trying to link distinguishable electrical brain activation patterns with specific mood-related symptoms, including symptoms of melancholic depression.

Several reviews have tried to summarize the findings in different ways. One narrative review from 2008 summarized HPA axis changes and sleep EEG in melancholic, respectively, atypical depression (3). However, limiting characteristics of this work was a lack of systematicity and absence of a methods section. Other EEG reviews covered tangential aspects, such as the potential of quantitative EEG as a biomarker and endophenotype in affective disorders in adults (4) or child psychiatric disorders (5), while a meta-analysis (6) and two narrative reviews (7, 8) covered electroencephalographic biomarkers as predictors of treatment response in major depressive disorder (MDD). One literature review focused on the role of quantitative EEG as a pharmacodynamic biomarker when developing new antidepressive drugs (9), while another focused on baseline EEG markers in MDD and attention deficit hyperactivity disorder (ADHD) (10). Three recent reviews of different methodological quality focused on frontal alfa asymmetry in MDD, but did not specifically address melancholic depression (11–13).

In summary, although EEG in the context of mood disorders has been subject to wide-ranging research, no systematic review has summarized the evidence of EEG as a potential biomarker in melancholic depression. Therefore, the purpose of this systematic review was to investigate whether electroencephalographic parameters can serve as a tool to distinguish between melancholic depression, non-melancholic depression, and healthy controls (HC) in adults. An introduction to the complexities of EEG theory and methodology is out of the scope of this systematic review; the aim was merely to systematically map the currently available literature, and as such, although strictly systematic in its conduction, it takes a “scoping” approach.

## Materials and Methods

### Registration, Reporting

The protocol adhered to the PRISMA-P statement (14) and was registered in the PROSPERO registry on August 8th, 2020, registration number CRD42020197472. The reporting was conducted according to PRISMA guidelines (15).

### Protocol Deviations

Two protocol deviations occurred: (1) DEX-CRH test was originally part of the search strategy but was abolished due to very few relevant studies (<5). (2) Due to the large degree of interstudy outcome diversity, we could not meaningfully perform the per protocol planned GRADE-assessment of each outcome.

### Information Sources and Search Strategy

Studies were identified by systematically searching the electronic databases PubMed, Embase, Google Scholar, and PsycINFO, using the following search strategy: melancholi^*^[Title/Abstract] OR endogeno^*^ depress^*^[Title/Abstract] OR “vital depression”[Title/Abstract]) AND (“EEG” OR electroencephalo^*^ OR electroencephalography [MeSH Terms]). Only English language papers were considered for inclusion. No publication date or publication status restrictions were imposed. To retrieve additional references, we performed a citation search (Web of Science) and scanned reference lists.

The chosen combination of databases was in line with a recent exploratory prospective study that concluded that this combination ensures an adequate and efficient coverage (16). The search strategy was developed in co-operation with a research librarian and information specialist. To detect unpublished studies, we searched for conference abstracts and the World Health Organization's clinical trials search portal (17).

## Eligibility

### Types of Studies

Clinical trials of all designs that performed an EEG-based examination on an adult patient group diagnosed with melancholic depression and compared with a control group of non-melancholic unipolar depressives and/or HC.

### Types of Participants

Participants aged +18y diagnosed with unipolar melancholic depression according to ICD, DSM, or another set of recognized diagnostic criteria. “Endogenous depression,” “endogenomorphic depression,” and “vital depression” was considered synonymic to melancholic depression. Additionally, studies with a subset of unipolar depressed patients described with a symptom cluster equivalent to melancholic features (i.e., unreactive mood, anhedonia, early morning awakening, psychomotor retardation, weight loss etc.) were eligible.

### Types of Intervention: Any EEG-Based Examination

Exclusion criteria: (1) animal studies, case reports, and reviews (2) studies with pediatric, adolescent, or exclusively elderly populations; (3) lack of relevant control group; (4) patients with psychotic or bipolar depression in the melancholic patient group (without relevant sub analysis); (5) participants suffering from comorbid illnesses likely to affect the EEG (e.g., epilepsy), or participants known with another major somatic/psychiatric illness.

### Selection Process

1. After eliminating duplicates, two independent reviewers (CFB, CJA) screened titles and abstracts to select the references eligible for full-text retrieval.
2. After full-text retrieval, the reviewers independently assessed the relevance of each by applying the inclusion criteria. This was done in an unblinded manner; i.e., the reviewers knew the authors' names, journal of publication, etc., when applying the criteria. Full texts that could not be retrieved electronically were sought for in university libraries and/or by direct contact to the authors via the internet. The full-text assessment for eligibility led to a final list of included primary studies in the systematic review.

The selection process was conducted using Endnote and Covidence for data management, with any disagreements resolved by consulting a senior reviewer (LVK).

### Data Extraction

Based on the Cochrane Consumers and Communication Review Group's data extraction template and The Strengthening of Reporting of Observational Studies in Epidemiology (STROBE) checklist (18, 19), we developed a data extraction sheet that listed the items to be extracted from each of the primary studies. Before the commencement of the data collection process, the data extraction sheet was pilot tested on ten random studies and refined accordingly. Two reviewers independently extracted data (CFB, CJA), i.e., the data extraction was done in duplicate and successively compared to eliminate errors and ensure validity. In the case of incongruity, a senior reviewer was consulted (LVK).

Acknowledging the concomitant lack of standardization in the reporting of EEG measures, methodological differences, and heterogeneity of studies, we took on a broad approach and defined EEG outcomes of interest as any EEG-based measure presented as a numeric value/score, e.g., a value representing the activity in any frequency band, frontal asymmetry/lateralization, a polysomnographic parameter, an event related potential component or any other EEG parameter or description.

### Risk of Bias of Individual Studies

We assessed the risk of bias at study level with the aim of giving each study appropriate weight when drawing conclusions. Since our pre-liminary literature search suggested that the published studies were non-randomized, and since the most appropriate study design for answering questions on diagnosis are case-control studies (20), we chose to use the STROBE checklist as an assessment tool (19).

STROBE is a 22-items reporting checklist covering cohort, case-control, and cross-sectional studies, developed by an international collaboration of epidemiologists, statisticians, and journal editors. Although not developed as a risk of bias tool, the checklist has proven useful in assessing key components of study quality in primary observational studies, facilitating a general judgement on the internal validity, as well as reflections on the risk of bias across studies, as previously shown by Teroganova et al. (21). Originally developed as a reporting guideline, the STROBE score also represents reporting transparency and comprehensibility.

Two reviewers (CFB, CJA) independently assessed the risk of bias of included studies using the STROBE checklist, reaching consensus in plenum if any disagreements occurred. Scores on the STROBE checklist were translated into a score (percentage), with scores ≥ 66% reflecting high study quality, ≤ 33 % low quality, and scores in between this range moderate quality. The STROBE scores were included in the Tables of Included Studies.

## Results

### Searches

**Figure 1:**
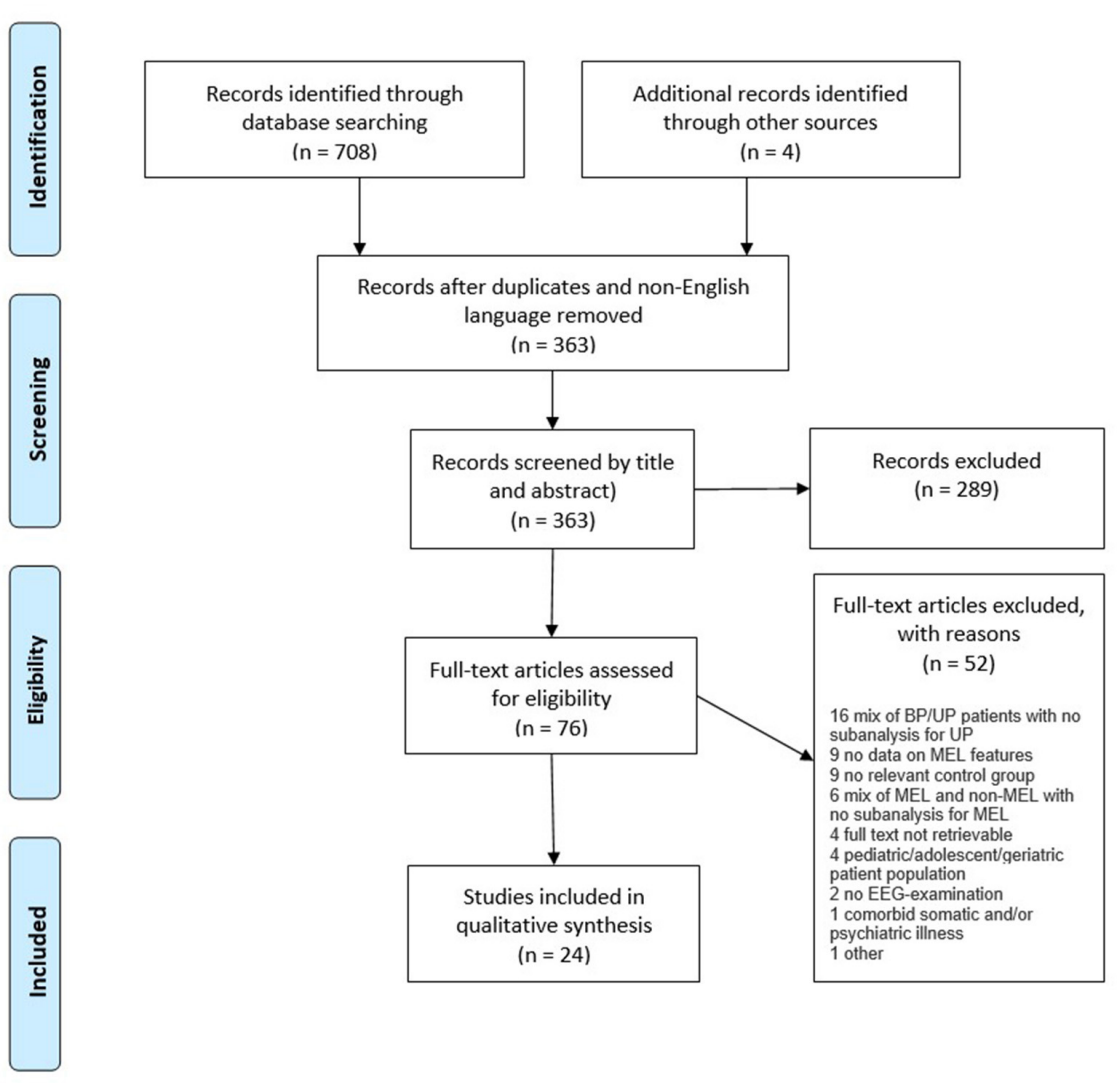
Study flow diagram.

An overview of the search procedures and study selection process (1–3) are presented in the PRISMA flow diagram (Figure 1). A total of 24 studies, all case-control designs, met the inclusion criteria. The captured studies performed a range of electroencephalographic interventions, which could be divided into three subgroups: Resting state studies (*n* = 5), sleep EEG studies (*n* = 10), and event-related potentials (ERP) studies (*n* = 9).

Of the 24 included studies, 21 could differentiate between melancholic depression, non-melancholic depression, and/or HC. By subgroup, four (22–25) out of five (22–26) resting state studies identified one or several EEG parameters that could distinguish groups; among the ERP studies, seven (27–33) out of nine (27–35) could separate melancholic depression from the other groups (27–33), while this was the case in all 10 sleep EEG studies (36–45).

Although sharing study design, the choice of EEG methods, analyses and outcome variables differed considerably within each subgroup, preventing any meaningful pooling of data to meta-analyses or any other statistical aggregation. Consequently, and according to our protocol, we proceeded with a narrative synthesis.

Regarding the quality of individual studies, 10 studies were classified as of low quality (22, 25, 28, 30, 31, 34, 36–38, 42), eight studies as of high quality (23, 24, 27, 29, 32, 33, 35, 40), and six studies of moderate quality (26, 39, 41, 43–45).

### General Information on the Summary of Studies Tables

#### Confounding Variables/Co-variates

This section covers any variable that was controlled for by either study design or in statistical analysis.

#### Choice of Nomenclature

For clarity, patients and control group(s) were labeled in a uniform way, so that all patient groups that met the inclusion criteria of the review were named melancholics (MEL) no matter the labeling in the origin paper (endogenous, endogenomorphic, melancholia, melancholic, etc.). Control groups of healthy controls (normal controls, healthy subjects, healthy volunteers etc.) were named HC, and non-melancholic unipolar MDD control groups were generally named non-MEL, except in the cases where authors specified another distinct MDD subtype.

#### Main Results

Main results with a significance level of 0.05 (or less) were included, i.e., no results at trend level were included. When no difference between groups was the main result, this was included in the table.

### Resting State Studies

Five studies performed a version of resting state EEG (22–26). Selected key characteristics of the studies are presented in Table 1A. Study sizes were generally small, with Quinn et al. differing by including as many as 57 patients with melancholic depression, 60 patients with non-melancholic depression, and 120 HC. One other study (23) also had dual control groups of patients with non-melancholic depression, while the three remaining studies (22, 25, 26) only had HC as a control group of relevance.

**Table 1A:** Summary of resting state studies.

**Study**	**Patients (%Female)**	**Diagnostic paradigm**	**Controls (%Female)**	**Method**	**Confounding variables, covariates**	**Main outcome variable(s)**	**Main results**	**Study quality (STROBE score in %)**	**Comments**
Ihl and Brinkmeyer (26)	22 (55%)	ICD-10	21 (62%) HC	300 s resting state EEG	Age, medication	Amount of beta activity, number of different segments	No differences between groups	Moderate (64%)	*In analysis, patient and control groups were sub grouped into a young and elderly group (age limits not specified)
Kano et al. (22)	21 (?)	RDC, DSM-III	44 (?) HC	30 sec. resting state EEG with spectral analysis	Age*, sex*, handedness	Topographical differences of alpha1, alpha2, beta1, and beta2 frequency bands	In MEL, alpha 2 was increased in the O1 area; beta 2 was increased at F4 and C4	Low (32%)	*Patients and controls were matched on age and sex, but not on subgroup level
Pizzagalli et al. (23)	20 (65%)	DSM-IV	18 (56%) HC + 18 (56%) non-MEL	Resting state EEG with LORETA analysis	Age, comorbid anxiety, depression severity, handedness, medication, sex, sociodemographic variables	3D intracerebral current density distribution for the delta, theta, alpha1, alpha2, beta1, beta2, and beta3 band	MEL had more activity than HC in right inferior frontal gyrus and less in the posterior cluster. Both MEL and non-MEL exhibited higher activity in the right frontal cluster than HC	High (82%)	
Quinn et al. (24)	57 (?)	DSM-IV	120 (?) HC + 60 (?) non-MEL	2 min. resting state EEG	Age*, depression severity, gender*, handedness*, medication, self-reported anxiety, and stress	EEG alpha asymmetry	Non-MEL displayed a relative global left-hemispheric activation relative to HC and MEL. MEL did not differ from HC	High (68%)	*Authors reported no significant differences between groups on these variables, but no crude numbers/means/*p*-values were reported
Zhang et al. (25)	10 (?)	CCMD-2	10 (?) HC	40 min. resting state EEG with wavelet entropy analysis	None/no information	Wavelet entropy values	Difference in wavelet entropy value between MEL and HC	Low (18%)	No raw data presented except for number of participants

Regarding eligibility criteria for the patient group, all studies reported diagnostic criteria [a version of ICD, DSM or the Chinese Classification of Mental Disorders (CCMD)]. The further specificity of inclusion and exclusion criteria varied; Pizzagalli et al. (23), Quinn et al., and Ihl et al. (23, 24, 26) stated specific inclusion and exclusion criteria for both patients and controls, while Kano et al. (22) were less detailed, although they were alone to depict an age range. Giving the least comprehensive insight into the inclusion process, Zhang et al. (25) reported that controls were “volunteers in a sober state.”

Regarding characteristics of participants, important data such as age (24, 25), medication status (24, 25), male and female sex (22, 24, 25), handedness (24–26), depression severity (22, 24–26), educational level (22–26), somatic comorbidity (22, 23, 25) and inpatient/outpatient status (22, 24, 25) were not reported.

Although all performing resting state EEG, the choice of methodology, analysis, and outcome variables of interest differed markedly between the five studies, as shown in Table 1A. Focusing on main results, four studies (22–25) reported significant differences in one or several EEG parameters between groups: While Quinn et al. (24) found that the non-melancholic group displayed a relative global left-hemispheric activation across frontal and parieto-temporal regions, but could not separate melancholics from HC, the results of the three remaining studies (22, 23, 25) revealed statistically significant differences between melancholics and HC: In a subgroup of 21 unipolar melancholic depressives, Kano et al. (22) analyzed the topographical differences of the alpha and beta frequency bands and found that alpha2 was statistically significantly increased in the O1 area and that beta2 was increased at F4 and C4 relative to HC. Performing whole-brain Low Resolution Electromagnetic Tomography (LORETA) analysis for alpha1, beta2, and beta3 frequency bands, Pizzagalli et al. (23) showed that melancholic subjects had more activity than HC in the right inferior frontal gyrus and less in the posterior cluster. Taking a different approach, Zhang et al. (25) used a nonlinear dynamics method based on wavelet entropy theory that, according to the authors, provided additional information compared to the frequency, amplitude, and energy measures of conventional EEG. Results of wavelet entropy analysis in resting state condition revealed that the melancholic group had greater wavelet entropy values than HC. With a STROBE-score of 18%, this study had weighty methodological problems, including no reporting of any participant characteristics and no mentioning of any attempts to address confounders in design or analysis.

### Event-Related Potential Studies (ERP Studies)

A total of nine studies investigated ERP components' ability to differentiate patients with melancholic features from patients with non-melancholic features and/or HC (27–35). Key characteristics are presented in Table 1B. Study sizes were generally rather small, with patient samples ranging from seven (34) to 60 (35) subjects. Two out of three studies (27, 29, 32–35) had control groups of both HC and non-melancholics, although the study by Kerr et al. (29) did not compare the two MDD groups. The remaining three studies had a single control group of HC (28, 30, 31).

**Table 1B:** Summary of ERP studies.

**Study**	**Patients (%Female)**	**Diagnostic paradigm**	**Controls (%Female)**	**Method**	**Confounding variables, covariates**	**Main outcome variable(s)**	**Main results**	**Risk of bias (STROBE score in %)**	**Comments**
Elton et al. (34)	7 (?) MEL	ICD-8, DSM-III	19 (63%) HC +21 (57%) reactive MDD	Auditory and visual EPs	None/no information	Early and late components of the CNV	No group differences in CNV measures	Low (27%)	
Fitzgerald et al. (27)	14 (71%) MEL	DSM-IV	16 (69%) HC + 13 (82%) non-MEL	Auditory EPs in resting state condition	Age, depression severity, medication	The slope of the N1/P2 (“IDAEP” slope)	The IDAEP slope was shallower in MELs compared to non-MEL and HC	High (68%)	
Gangadhar et al. (28)	17 (64%) MEL	DSM-IIIR	22 (59%) HC	Auditory oddball EPs	Age, depression severity*, medication, sex	P300 amplitudes and latencies	Pre-treatment P300 amplitudes in MEL were smaller than in HC. No difference between Mel and HC in pre-treatment P300 latencies.	Low (32%)	*Authors mention that pre-treatment P300 amplitudes in MELs were smaller and negatively correlated with severity, but no crude HRSD scores were reported
Kerr et al. (29)	49 (67%) MEL	M.I.N.I*	98 (56%) HC + 34 (50%) non-MEL**	Auditory oddball EPs with deconvolution analysis and model-based fitting	Age, depression severity, medication, sex	Deconvolution measures (peak area and latency), five parameters of a thalamocortical model of neuronal activity	MEL were found to have increased thalamocortical transmission delays compared to HC, with the size of the increase strongly correlated with depression severity	High (68%)	*Not specified whether DSM-IV and/or ICD-10 criteria were used **No comparison between MEL and non-MEL
Khanna et al. (29)	30 (47%) MEL	DSM-III	40 (35%) HC	Auditory and visual EPs	Medication	Latencies and amplitudes for P1, N1, and P2 components	No differences between groups	Low (27%)	
Khanna et al. (29)	26 (42%) MEL	DSM-III	26 (42%) HC	Bilateral movement of thumbs in response to changes on an oscilloscope screen	Sex, medication	Latency and amplitude of the pre-motor potential	Lower pre-motor potential amplitude in MEL	Low (27%)	
Liu et al. (32)	38 (61%) MEL	DSM-IV	113 (66%) HC + 103 (69%) non-MEL	Reward anticipation task involving two conditions	Age, co-morbid anxiety, depression severity, handedness, medication, sex, study location	Frontal EEG asymmetry scores	Melancholic symptoms* was associated with frontal EEG asymmetry during reward anticipation independent of depression severity	High (82%)	*When defined dimensionally
Quinn et al. (35)	60 (63%) MEL	DSM-IV	114 (61%) HC + 54 (43%) non-MEL	Visual cognitive Go/No-Go task.	Age, anxiety, depression severity, medication, psychomotor disturbances, sex	Amplitude and latency of the P200, N200, and P300 component	No differences between groups	High (68%)	
Shankman et al. (34)	17 (53%) MEL	DSM-IV	34 (71%) HC + 48 (71%) non-MEL	Computerized slot-machine game with a pre- and post-goal phase involving two conditions	Age, depression severity, employment status, handedness, medication, sex	EEG asymmetry for frontal and posterior alpha power	Post-goal posterior asymmetries differed between MEL and non-MEL	High (86%)	

Regarding eligibility criteria, four studies (27, 28, 32, 35) reported explicit criteria for both patients and control groups(s). Of these, only two studies reported age limits as part of the inclusion criteria (28, 32). Three studies were less specific, reporting an ICD/DSM-diagnosis for the eligible patients, but otherwise giving loose or very brief criteria, not reporting inclusion/exclusion criteria for the control group(s) (30, 31, 34). One study referred to another publication for eligibility criteria (33).

Concerning clinical and demographic characteristics of participants, all studies lacked data on one or several variables, namely medication status (34), male/female sex (34), handedness (27, 29–31, 34, 35), socioeconomic status (e.g., years of education, employment status) (27–32, 34, 35), inpatient/outpatient status (27–33, 35), comorbidity (34) and depression severity (28, 30, 31, 34). All studies, except one for one (34), included information on participants' medication status. The choice of “event” differed among studies, with an overweight of sensory stimulation (e.g., a visual stimulus, an auditory stimulus), in some cases combined with a motor event (e.g., pressing a button) or involving a mental operation (e.g., anticipation). In several studies, the experimental setup involved two conditions, an incentive and a non-incentive.

Two studies examined ERP components indicating preparatory activity prior to a behavior, namely the so-called Bereitschaftspotential (BP) or pre-motor potential (31) and the Contingent Negative Variation (CNV) potential (34). As the terminal CNV resembles the BP, some researchers have claimed that they are the same component. Khanna et al. (31) found lower BP amplitude in melancholics compared to HC, while Elton et al. (34) in a sample size comprising seven melancholic patients, found no differences between melancholics, reactive MDD, and HC.

In four studies, ERPs of auditory stimuli were examined (27–30). In two of these, traditional odd-ball paradigms, where sequences of repetitive stimuli were infrequently interrupted by a deviant stimulus, eliciting a transient activity in prefrontal cortical regions, were core elements of the study designs (28, 29). Analyzing the classic P300 component, Gangadhar et al. (28) found smaller amplitudes in melancholics compared to HC, but no differences in latencies. The complex design of Kerr et al. (29) involved deconvolution analysis and fitting data to a neuronal transmission activity model, leading to the result that melancholics were found to have increased thalamocortical transmission delays compared to HC, with the size of the increase strongly correlated with depression severity. Using the intensity dependence of the auditory evoked potential (IDAEP), an ERP measure regarded as a reliable indicator of central serotonin function in depression, Fitzgerald et al. (27) could distinguish patients with melancholic depression from patients with non-melancholic depression and HC, while Khanna et al. (30) found no significant differences between groups in a study investigating both auditory and visually evoked potentials. Concentrating on visual stimuli in a cognitive go/no-go task, Quinn et al. (35) also failed to separate patients with melancholic depression from control groups when comparing amplitude and latency of the P200, N200, and P300 components.

Hypothesizing that melancholic depression is characterized by a blunted response to reward, two studies with overlapping author groups examined deficits in reward processing, both measuring EEG asymmetry during a behavioral task that elicited reward processing (32, 33): Liu et al. (32) found melancholic symptoms when measured dimensionally (but not categorically) to be associated with frontal EEG asymmetry during reward anticipation independent of depression severity, while Shankman et al. (33) found that post-goal posterior (but not frontal) asymmetry differed between melancholic and non-melancholic patients.

### Sleep EEG Studies

Ten of the included studies performed sleep EEG (36–45). Key characteristics are presented in Table 1C. Study sizes ranged from eight (40) to 75 (39) patients, and the choice of control group(s) and specifics of the setup varied markedly. Of relevance to this review, half of the studies had HC as the only control group (36, 37, 42, 43, 45), and two studies had a single control group of non-melancholic MDD (40, 44). The remaining three studies had two control groups that met inclusion criteria; two had HC and non-melancholic MDD patients (39, 41), while Frank et al. (38) as the only study assessed endogenous origin of depression and melancholic symptomatology individually, creating three subgroups of MDD patients, namely endogenous-melancholics, endogenous non-melancholics, and non-endogenous non-melancholics.

**Table 1C:** Summary of sleep EEG studies.

**Study**	**Patients (%F)**	**Diagnostic paradigm**	**Controls (%F)**	**Method**	**Confounding variables, covariates**	**Main outcome variable(s)**	**Main results**	**Risk of bias (STROBE score in %)**	**Comments**
Dippel et al. (36)	10 (?) MEL	no data	10 (?) HC	Three nights of sleep EEG with analysis of data from the 3rd night	Age, medication, sex	Latency, density and duration of 1st REM period	Mean density of REM was increased in MEL	Low (14%)	
Feinberg et al. (37)	18 (72%) MEL*	RDC	41 (51%) HC*	Two nights of sleep EEG data were used to derive discriminant functions (DFs). The DFs were validated using sleep EEG data from another patient sample	Medication	Discriminant functions comprising traditional sleep EEG variables	A 5 variable (REM latency and 4 sleep continuity measures), two-group discriminant function classified 76% of MEL and HC correctly.	Low (27%)	*The two independent patient samples were mixed uni/bipolar MEL, but with subanalysis for UP group. Clinical data were only partially reported for the UP subgroup and controls. We report here available data.
Frank et al. (38)	38 (76%) MEL	RDC	46 (67%) endogenous non-MEL 44 + (86%) non-endogenous non-MEL*	Sleep EEG (not specified)	Age, depression severity, medication	13 EEG sleep variables, divided into sleep continuity, sleep architecture, and REM sleep indices	Sleep efficiency differentiated the groups, with MEL showing the poorest sleep efficiency and the non-endogenous showing the best	Low (32%)	*Study including only MDD patients, where endogenous origin of depression and melancholic symptomatology were separated
Giles et al. (39)	75 (?)* MEL	RDC	54 (59%) HC + 102 (?)* non-MEL	Two nights sleep EEG with no habituation night	Age, depression severity, inpatent/outpatient status, medication, sex	Mean REM latencies	MEL had lower mean REM latency than non-MEL, and both groups had lower mean REM latencies than HC	Moderate (50%)	*No data for MEL and non-MEL subgroups
Hein et al. (40)	8 (?)* MEL	DSM-IV-TR	8 (?)* reactive MDD	Three consecutive nights in sleep laboratory with analysis of data from night two or three	Age, depression severity, medication	A selection of sleep variables + network organization parameters during REM and Slow Wave Sleep (SWS)	No difference between MEL and reactive MDD for polysomnographic data. For network organization parameters, MEL showed an increase in the small-world coefficient during REM for the delta band compared to reactive MDD	High (82%)	*No data for MEL and reactive MDD subgroups
Hubain et al. (41)	15 (0%) MEL	RDC	20 (0%) HC + 24 (0%) non-MEL	Three consecutive nights of sleep EEG. Only data from the 3rd night was considered for analysis	Age, depression severity, medication, sex	A selection of sleep EEG variables	REM latency <50 min was more frequently observed in MEL compared to non-MEL. Compared to HC, MEL showed shortened REM latency, reduced Small Wave Sleep and reduced stage II.	Moderate (64%)	
Iorio et al. (42)	10 (90%) MEL	RDC	10 (90%) HC	Three consecutive nights of sleep EEG	Medication, sex	A selection of sleep variables + transition variables (transitions from each stage to any other one, number of stage occurrences, stage durations)	HC had larger values of total sleep time, sleep efficacy, and REM in 2nd half of sleep, while MEL had larger values of awake time and REM in 1st half of sleep. The probability of transition from stage 1 to stage 2 were higher in HC, while the probability of transition from stage 1 to awake was lower.	Low (32%)	
Lange (43)	34 (?) MEL	No data*	16 (?) HC	Two nights of sleep EEG. Only data from the 2nd night was considered for analysis	None/no information	REM cycle duration, REM latency, spectral EEG analysis parameters	MEL showed decreased differentiation between synchronized and desynchronized states during sleep and wakefulness and a slowing of ultradian cycle during early morning hours. No differences in REM cycle duration or REM latency between groups	Moderate (50%)	*A diagnosis of vital depression based on 3 criteria: (1) limited occurrence of depressed phases separated by remission intervals; (2) significantly detectable diurnal variations of mood with the highest intensity of depressive symptoms in the early morning; (3) self-reliant depressed episodes together with lack of social reason to explain the phasic occurrence and diurnal variation of mood.
Rush et al. (44)	32 (66%) MEL	RDC	38 (66%) non-MEL	Two consecutive nights of sleep EEG	Age, depression severity, medication	A selection of sleep EEG variables	MEL had lower mean REM latency, non-REM sleep time, total sleep time, and stage 2 time	Moderate (55%)	
Sitaram et al. (45)	20 (?) MEL	RDC	26 (?) HC	Four nights of sleep EEG including two nights with a pharmacologic challenge* during night three and four	Medication	A selection of baseline sleep EEG variables and arecoline response (time from infusion to second REM episode), with discriminant function analysis	MEL had reduced REM latency, arecoline response, delta sleep, and increased density of 1st REM period, total REM density, intermittent awake time, early morning awake time and REM%	Moderate (45%)	*Administration of 1) glycopyrrolate 0.15 mg and 2) arecoline 0.5 mg or placebo was administered at the end of the first REM period.

Four studies reported sufficient eligibility criteria for patients (37, 38, 40, 44), while the remaining studies stated a diagnosis but were otherwise unspecific (42, 45) or did not mention the used diagnostic paradigm (36, 43). In defining eligibility for control group(s), three studies gave quite precise criteria (38, 40, 41, 44), two were less specific (37, 45), and another three studies gave none or very sparse criteria (36, 37, 42, 43). Most studies did not mention age limits as part of the eligibility criteria (37–39, 41).

Information for patients and/or controls on several demographic or clinical variables were not reported, including age (36, 39, 45), male/female sex (36, 40, 43, 45), handedness (36–45), depression severity (36, 37, 39, 42, 43), educational level (36–45), somatic comorbidity (36, 42, 43, 45) and inpatient/outpatient status (36, 37, 40, 42, 43, 45). All studies included information on participants' medication status.

Concerning methodology, half of the studies reported simultaneous monitoring of electrooculography (EOG) and electromyography (EMG) (39, 40, 42, 44, 45). One study (38) only recorded EOG, while four studies (36, 37, 41, 43) did not record EMG and EOG (or did not report doing so), probably exposing their raw data to more artifacts. Four studies reported having a habituation night to avoid “first night effects” on sleep EEG parameters (36, 41, 43, 45), while the six remaining studies did not report doing this (37–39, 42).

Again, a high level of methodological variability was present, but as a common trait, all studies found one or several parameters that could distinguish melancholics from control group(s):

Several studies found shortened REM latency in melancholics compared to non-melancholics (39, 41, 44) and HC (39, 41, 45). Together with four sleep continuity measures, REM latency took part in a five variable, two-group discriminant function that classified 35 out of 46 (76%) of MEL and HC subjects correctly (37). Regarding REM density, two studies found increased REM density in patients with melancholic depression compared to HC (36, 45). Two studies reported the total amount of REM sleep in patients with melancholic depression compared to HC: In one study, REM sleep was reported as a percentage of total sleep time and found elevated in the melancholic group (45), while the other study found total REM to be elevated in the first half of the night in HC compared to the melancholic group, and vice versa in the second half (42). However, not all studies found significant differences in REM sleep parameters (38, 40, 43). Unsurprisingly, patients with melancholic depression were found to have larger values of total sleep time (37, 42, 44) and sleep efficacy (a ratio of time spent asleep/total recording period X 100) (37, 38, 42), increased intermittent awake time and earlier morning awake time (45) compared to control groups. Tapping into the complex theory of network organization, Hein et al. found that for network organization parameters, melancholics showed an increase in the so-called small-world coefficient during REM for the delta band compared to a control group of reactive MDD (40). In a study performing spectral EEG analysis, melancholics showed decreased differentiation between synchronized and desynchronized states during sleep and wakefulness and a slowing of an ultradian cycle during early morning hours (43).

## Discussion

Despite limitations, the general trend of studies identified in this systematic review was that multiple EEG modalities showed an ability to distinguish melancholic patients from non-melancholic patients and/or HC, highlighting electroencephalography as a potential non-invasive, low-cost real-time potential biomarker for melancholic depression. In the following, the advantages and limitations of the review will be discussed.

### Advantages and Limitations

In the context of this review, the STROBE score was interpreted as reflecting study quality, but one should keep in mind that it was constructed as a reporting guideline; thus, one can imagine studies with methodological difficulties, but with meticulous reporting, obtaining a high STROBE score (and vice versa). Since many of the low-quality studies were published in the early 80's to early 90's with a tendency toward higher STROBE scores in younger publications, increased streamlining and improvement in reporting in recent years may play a part. Noteworthy, all STROBE items weigh equally when calculating the final score, and the tool does not consider all parameters, e.g., sample size. As such, STROBE scores should be interpreted cautiously and in the context of the additional information in the tables and main text, but the general picture of methodological difficulties of the included studies remains intact, with the note that most studies rated of moderate-high quality also were prone to bias.

An inevitable source of bias was the shifting diagnostic paradigms; especially the inclusion of older studies may have contributed to this by not always separating endogenous patients in unipolar and bipolar subgroups. This we addressed by excluding studies that did not separate unipolar and bipolar patients in the analysis. A key problem lies in the methods used to diagnose “true melancholia” and differentiate it from non-melancholic depression, with many of the studies reviewed using criteria that may not have ensured such a distinction. On the other hand, studies published in the 80s and early 90s could be considered of higher quality at the diagnostic level, especially those using RDC criteria, as RDC criteria by many are considered closer to delimit “true melancholia” from non-melancholic (reactive) depression (46). In addition, the pragmatic choice of treating endogenous and melancholic as synonymous can be problematized as “endogenous” traditionally may imply an absence of a triggering cause (47, 48).

Publication bias cannot be excluded and is difficult to evaluate due to the heterogeneous EEG methods, analyses, and outcome variables, preventing meaningful pooling of data in a meta-analysis. At the review-level, reporting bias was addressed by the protocol registration and PRISMA reporting of the review, as outlined in the methods section. Selection bias from missed studies was minimized by a comprehensive search for all published studies across multiple databases, including reduction of selection bias due to non-publication by searching conference abstracts and clinical trial registries. As outlined in the PRISMA flow diagram, four potentially eligible studies could not be retrieved despite extensive efforts, including repeated contact attempts to the authors.

The heterogeneity in findings was not surprising in the light of the marked variability on almost all levels across studies, preventing pooling of data and a formal statistical investigation of heterogeneity. However, although there was a degree of inconsistency in the results, the overall picture was no effects in complete opposite directions or large variations in the effect(s) on the outcome(s); all the potential modifiers such as methodological characteristics, subpopulations, intervention components and contextual factors aside, the general trend was, that multiple EEG modalities showed an ability to distinguish patients with a specific symptom profile of neurovegetative symptoms. This was exemplified in the sleep eeg studies, where—regardless of analyzing traditional sleep variables, performing a group comparison of background activity with spectral analysis, computing network organization parameters or another method—all studies found one or several parameters that could distinguish between patients with melancholic depression from control group(s), with a clustering around different aspects of REM sleep. Interestingly, these results echoed the conclusion of a non-systematical review from 1982 (49) that highlighted reduced REM latency, increased REM density, reduction in delta sleep, and impaired sleep efficiency as possible melancholia biomarkers, commenting that “sleep EEGs are pragmatically difficult, but results are quite specific.” However, several significant confounders cloud the picture: Firstly, certain antidepressants (e.g., tricyclics) are known to suppress REM sleep, possibly evoking a rebound effect explaining the decreased REM latency (50, 51). Secondly, even in unmedicated subjects, the fact that melancholic depression is associated with early morning wakening could also ignite a rebound effect (i.e., decreased REM latency), as percent of time spend in REM sleep increases during the night. A pattern similar to the sleep studies, although less obvious, could be seen in the ERP and resting state studies; although the first group performed quite different interventions, most studies did find differences between melancholics and control group(s) in various evoked potential components, providing evidence for differences in neuronal processing in patients diagnosed with melancholic depression. As for the smallest study group, the resting state studies, the same tendency was present, although conclusions were hampered by the small study number (*n* = 5) and the low quality of especially one study (25).

## Conclusion

Covering publications across a span of almost 40 years, the included studies were subject to clinical and methodological heterogeneity, preventing aggregation of data. Studies were challenged on several aspects on an individual level, such as susceptibility to risk of information and selection bias, low statistical power due to small samples, and not considering possible confounders in analysis. However, all limitations aside, the general trend was that multiple EEG modalities showed an ability to distinguish melancholic patients from non-melancholic patients and/or HC. Being non-invasive, low-cost, yet offering real-time information about neuronal oscillations, and with the prospect of integrating new modeling techniques, electroencephalography remains a candidate modality for a clinically useful biomarker for melancholic depression.

## Data Availability Statement

The raw data supporting the conclusions of this article will be made available by the authors, without undue reservation.

## Author Contributions

LK designed the study together with CB. CB and CA conducted the data extraction. CB wrote the first draft of the manuscript that was revised by CA and LK. LK had full access to all the data in the study and takes responsibility for the integrity of the data and the accuracy of the data analysis. All authors contributed to the article and approved the submitted version.

## Conflict of Interest

LK has been a consultant for Lundbeck and Teva for the last three years. The remaining authors declare that the research was conducted in the absence of any commercial or financial relationships that could be construed as a potential conflict of interest.

## Publisher's Note

All claims expressed in this article are solely those of the authors and do not necessarily represent those of their affiliated organizations, or those of the publisher, the editors and the reviewers. Any product that may be evaluated in this article, or claim that may be made by its manufacturer, is not guaranteed or endorsed by the publisher.

## References

[1] American Psychiatric Association AP. Diagnostic and Statistical Manual of Mental Disorders (5th ed.). Arlington, VA, Wasington, DC: American Psychiatric Association (2013). 10.1176/appi.books.9780890425596

[2] World Health Organization. International Classification of Diseases, 11th revision. The World Health Organization. Available online at: https://icd.who.int/en (accessed October 1, 2020).

[3] Antonijevic I. HPA axis and sleep: identifying subtypes of major depression. Stress. (2008) 11:15–27. 10.1080/10253890701378967 17853067

[4] Kesebir S Yosmaoglu A. QEEG in affective disorder: about to be a biomarker, endophenotype and predictor of treatment response. Heliyon. (2018) 4:e00741. 10.1016/j.heliyon.2018.e00741 30148219 PMC6106696

[5] McVoy M Lytle S Fulchiero E Aebi ME Adeleye O Sajatovic M. A systematic review of quantitative EEG as a possible biomarker in child psychiatric disorders. Psychiatry Res. (2019) 279:331–44. 10.1016/j.psychres.2019.07.004 31300243

[6] Widge AS Bilge MT Montana R Chang W Rodriguez CI Deckersbach T. Electroencephalographic biomarkers for treatment response prediction in major depressive illness: a meta-analysis. Am J Psychiatry. (2019) 176:44–56. 10.1176/appi.ajp.2018.17121358 30278789 PMC6312739

[7] Schiller MJ. Quantitative electroencephalography in guiding treatment of major depression. Front Psychiatry. (2018) 9:779. 10.3389/fpsyt.2018.00779 30728787 PMC6351457

[8] Wade EC Iosifescu DV. Using electroencephalography for treatment guidance in major depressive disorder. Biol Psychiatry Cogn Neurosci Neuroimaging. (2016) 1:411–22. 10.1016/j.bpsc.2016.06.002 29560870

[9] Jones KA Menniti FS Sivarao DV. Translational psychiatry–light at the end of the tunnel. Ann N Y Acad Sci. (2015) 1344:1–11. 10.1111/nyas.12725 25752480

[10] Olbrich S van Dinteren R Arns M. Personalized medicine: review and perspectives of promising baseline EEG biomarkers in major depressive disorder and attention deficit hyperactivity disorder. Neuropsychobiology. (2015) 72:229–40. 10.1159/000437435 26901357

[11] Fernandez-Palleiro P Rivera-Baltanas T Rodrigues-Amorim D Fernandez-Gil S del Carmen Vallejo-Curto M Alvarez-Ariza M. Brainwaves oscillations as a potential biomarker for major depression disorder risk. Clin EEG Neurosci. (2020) 51:3–9. 10.1177/1550059419876807 31537100

[12] Kaiser AK Gnjezda MT Knasmuller S Aichhorn W. Electroencephalogram alpha asymmetry in patients with depressive disorders: Current perspectives. Neuropsychiatric Dis Treat. (2018) 14:1493–504. 10.2147/NDT.S137776 29928121 PMC6001846

[13] van der Vinne N Vollebregt MA van Putten M Arns M. Frontal alpha asymmetry as a diagnostic marker in depression: fact or fiction? A meta-analysis. Neuroimage Clin. (2017) 16:79–87. 10.1016/j.nicl.2017.07.006 28761811 PMC5524421

[14] Moher D Shamseer L Clarke M Ghersi D Liberati A Petticrew M. Preferred reporting items for systematic review and meta-analysis protocols (PRISMA-P) 2015 statement. Syst Rev. (2015) 4:1. 10.1186/2046-4053-4-1 25554246 PMC4320440

[15] Liberati A Altman DG Tetzlaff J Mulrow C Gotzsche PC Ioannidis JP. The PRISMA statement for reporting systematic reviews and meta-analyses of studies that evaluate healthcare interventions: explanation and elaboration. BMJ. (2009) 339:b2700. 10.1136/bmj.b2700 19622552 PMC2714672

[16] Bramer WM Rethlefsen ML Kleijnen J Franco OH. Optimal database combinations for literature searches in systematic reviews: a prospective exploratory study. Syst Rev. (2017) 6:245. 10.1186/s13643-017-0644-y 29208034 PMC5718002

[17] The World Health Organization's Clinical Trials Search Portal. Available online at: https://apps.who.int/trialsearch/ (accessed October 1, 2020).

[18] Ryan R SA Prictor M Hill S. Cochrane Consumers and Communication Group Data Extraction Template for Included Studies (2016). Available online at: http://cccrg.cochrane.org/author-resources.

[19] von Elm E Altman DG Egger M Pocock SJ Gøtzsche PC Vandenbroucke JP. The Strengthening the Reporting of Observational Studies in Epidemiology (STROBE) statement: guidelines for reporting observational studies. PLoS Med. (2007) 4:e296. 10.1371/journal.pmed.0040296 17941714 PMC2020495

[20] Ryan R HS Broclain D Horey D Oliver S Prictor M. Cochrane Consumers and Communication Review Group. Study Design Guide (2013). Available online at: http://cccrg.cochrane.org/author-resources (accessed October 1, 2020).

[21] Teroganova N Girshkin L Suter CM Green MJ. DNA methylation in peripheral tissue of schizophrenia and bipolar disorder: a systematic review. BMC Genet. (2016) 17:27. 10.1186/s12863-016-0332-2 26809779 PMC4727379

[22] Kano K Nakamura M Matsuoka T Iida H Nakajima T. The topographical features of EEGs in patients with affective disorders. Electroencephalogr Clin Neurophysiol. (1992) 83:124–9. 10.1016/0013-4694(92)90025-D 1378377

[23] Pizzagalli DA Nitschke JB Oakes TR Hendrick AM Horras KA Larson CL. Brain electrical tomography in depression: the importance of symptom severity, anxiety, and melancholic features. Biol Psychiatry. (2002) 52:73–85. 10.1016/S0006-3223(02)01313-6 12113998

[24] Quinn CR Rennie CJ Harris AW Kemp AH. The impact of melancholia versus non-melancholia on resting-state, EEG alpha asymmetry: electrophysiological evidence for depression heterogeneity. Psychiatry Res. (2014) 215:614–7. 10.1016/j.psychres.2013.12.049 24467874

[25] Zhang S Qiao S. Analysis of EEG in melancholia based on wavelet entropy and complexity. In: 2010 4th International Conference on Bioinformatics and Biomedical Engineering. Chengdu (2010). p. 1–4. 10.1109/ICBBE.2010.5515955

[26] Ihl R Brinkmeyer J. Differential diagnosis of aging, dementia of the Alzheimer type and depression with EEG-segmentation. Dement Geriatr Cogn Disord. (1999) 10:64–9. 10.1159/000017103 10026377

[27] Fitzgerald PB Mellow TB Hoy KE Segrave R Cooper NR Upton DJ. A study of intensity dependence of the auditory evoked potential (IDAEP) in medicated melancholic and non-melancholic depression. J Affect Disord. (2009) 117:212–6. 10.1016/j.jad.2009.01.009 19201033

[28] Gangadhar BN Ancy J Janakiramaiah N Umapathy C. P300 amplitude in non-bipolar, melancholic depression. J Affect Disord. (1993) 28:57–60. 10.1016/0165-0327(93)90077-W 8326081

[29] Kerr CC Kemp AH Rennie CJ Robinson PA. Thalamocortical changes in major depression probed by deconvolution and physiology-based modeling. Neuroimage. (2011) 54:2672–82. 10.1016/j.neuroimage.2010.11.008 21073966

[30] Khanna S Mukundan CR Channabasavanna SM. Middle latency evoked potentials in melancholic depression. Biol Psychiatry. (1989) 25:494–8. 10.1016/0006-3223(89)90204-7 2930813

[31] Khanna S Mukundan CR Channabasavanna SM. Bereitschaftspotential in melancholic depression. Biol Psychiatry. (1989) 26:526–9. 10.1016/0006-3223(89)90073-5 2790093

[32] Liu H Sarapas C Shankman SA. Anticipatory reward deficits in melancholia. J Abnorm Psychol. (2016) 125:631–40. 10.1037/abn0000172 27175986 PMC4925246

[33] Shankman SA Sarapas C Klein DN. The effect of pre- vs. post-reward attainment on EEG asymmetry in melancholic depression. Int J Psychophysiol. (2011) 79:287–95. 10.1016/j.ijpsycho.2010.11.004 21111010 PMC3038177

[34] Elton M. A longitudinal investigation of event-related potentials in depression. Biol Psychiatry. (1984) 19:1635–49. 6518213

[35] Quinn CR Harris A Kemp AH. The impact of depression heterogeneity on inhibitory control. Aust N Z J Psychiatry. (2012) 46:374–83. 10.1177/0004867411432073 22508597

[36] Dippel B Lauer C Riemann D Majer-Trendel K Krieg J-C Berger M. Sleep and dreams in eating disorders. Psychotherap Psychosomat. (1987) 48:165–9. 10.1159/000288048 3505710

[37] Feinberg M Gillin JC Carroll BJ Greden JF Zis AP. EEG studies of sleep in the diagnosis of depression. Biol Psychiatry. (1982) 17:305–16. 7082698

[38] Frank E Kupfer DJ Hamer T Grochocinski VJ McEachran AB. Maintenance treatment and psychobiologic correlates of endogenous subtypes. J Affect Disord. (1992) 25:181–9. 10.1016/0165-0327(92)90004-P 1527273

[39] Giles DE Roffwarg HP Rush AJ Guzick DS. Age-adjusted threshold values for reduced REM latency in unipolar depression using ROC analysis. Biol Psychiatry. (1990) 27:841–53. 10.1016/0006-3223(90)90465-E 2331494

[40] Hein M Lanquart J-P Loas G Hubain P Linkowski P. Alterations of neural network organisation during rapid eye movement sleep and slow-wave sleep in major depression: implications for diagnosis, classification, and treatment. Psychiatry Res Neuroimaging. (2019) 291:71–8. 10.1016/j.pscychresns.2019.08.003 31416044

[41] Hubain PP Souery D Jönck L Staner L Van Veeren C Kerkhofs M. Relationship between the Newcastle scale and sleep polysomnographic variables in major depression: a controlled study. Eur Neuropsychopharmacol. (1995) 5:129–34. 10.1016/0924-977X(95)00011-D 7549455

[42] Iorio G Marciano F Martino M Kemali D. Statistical comparison of transition sleep variables in depressed and normal subjects. Europ Psychiatry. (1994) 9:95–100. 10.1017/S0924933800001826

[43] Lange H. EEG spectral analysis in vital depression: ultradian cycles. Biol Psychiatry. (1982) 17:3–21. 6120724

[44] Rush AJ Giles DE Roffwarg HP Parker CR. Sleep EEG and dexamethasone suppression test findings in outpatients with unipolar major depressive disorders. Biol Psychiatry. (1982) 17:327–41. 7082700

[45] Sitaram N Dube S Jones D Pohl R Gershon S. Acetylcholine and alpha 1-adrenergic sensitivity in the separation of depression and anxiety. Psychopathology. (1984) 17(Suppl. 3):24–39. 10.1159/000284129 6505119

[46] Türkçapar MH Akdemir A Orsel SD Demirergi N Sirin A Kiliç EZ. The validity of diagnosis of melancholic depression according to different diagnostic systems. J Affect Disord. (1999) 54:101–7. 10.1016/S0165-0327(98)00146-3 10403153

[47] Kessing LV. Epidemiology of subtypes of depression. Acta Psychiatr Scand Suppl. (2007) 115:85–9. 10.1111/j.1600-0447.2007.00966.x 17280574

[48] Kessing LV. Endogenous, reactive and neurotic depression – diagnostic stability and long-term outcome. Psychopathology. (2004) 37:124–30. 10.1159/000078611 15153744

[49] Greden JF. Biological markers of melancholia and reclassification of depressive disorders. Encephale. (1982) 8:193–202. 6809444

[50] McCarthy A Wafford K Shanks E Ligocki M Edgar DM Dijk DJ. REM sleep homeostasis in the absence of REM sleep: effects of antidepressants. Neuropharmacology. (2016) 108:415–25. 10.1016/j.neuropharm.2016.04.047 27150557

[51] Vogel GW Buffenstein A Minter K Hennessey A. Drug effects on REM sleep and on endogenous depression. Neurosci Biobehav Rev. (1990) 14:49–63. 10.1016/S0149-7634(05)80159-9 1970148

